# Journal of Neonatal Surgery: Now on PubMed

**Published:** 2015-07-01

**Authors:** Bilal Mirza, Yogesh Kumar Sarin

**Affiliations:** 1 Department of Pediatric Surgery, The Children’s Hospital and the Institute of Child Health, Lahore, Pakistan; 2 Department of Pediatric Surgery Maulana Azad Medical College New Delhi, India

It is a formidable task to start a journal about a discipline that doesn’t even exist as a separate specialty in most parts of the world till date. Further, the journal was the vision of few like-minded intellectuals who neither represented any professional body or society, nor had any means to raise funds for this endeavor. But they had energy and optimism to follow their dream. When we pondered over the high morbidity and mortality of surgical neonates, we realized that the possible solutions lie in evidence based practice, training of pediatric and neonatal surgery health care staff, and reduction of preventable morbidity and mortality. Launching the Journal of Neonatal Surgery is also a humble step towards this endeavor. We believe that this has given Neonatal Surgery a special status within the gamut of Pediatric Surgery and would give impetus to the establishment of a separate Neonatal Surgery Health Services. Appreciation and recognition of one’s work is the fuel for further hike. We started this project with a vision that if not success, the intention and struggle are in our control. It is a splendid moment for us to announce its achievements as indicated by its wide readership. Its wide penetration is also reflected in the global contribution of the manuscripts. Indexing of the Journal of Neonatal Surgery with PubMed and PubMed Central is an additional feather in its cap. All the manuscripts published since inception of Journal of Neonatal Surgery have been archived in PubMed Central and they can be retrieved free of cost. This will definitely further increase our visibility and readership. Indexing with Medline and getting Impact Factor are our next goals, albeit we have been calculating our unofficial impact factor. All these attainments would have not been possible without contributions from our authors, peer reviewers, and editorial team members and we gratefully acknowledge them for their continuous support for sustaining this open access venture.

## Footnotes

**Source of Support:** Nil

**Conflict of Interest:** Both the authors are editors of the journal. This article is not externally peer reviewed.

